# Enhancing the inhibition of dental erosion and abrasion with quercetin-encapsulated hollow mesoporous silica nanocomposites

**DOI:** 10.3389/fbioe.2024.1343329

**Published:** 2024-02-08

**Authors:** Jia-Min Chen, Yi-Ling Cheng, Meng-Hui Yang, Chen Su, Hao Yu

**Affiliations:** ^1^ Fujian Key Laboratory of Oral Diseases and Fujian Provincial Engineering Research Center of Oral Biomaterial and Stomatological Key Laboratory of Fujian College and University, School and Hospital of Stomatology, Fujian Medical University, Fuzhou, China; ^2^ Department of Prosthodontics, School and Hospital of Stomatology, Fujian Medical University, Fuzhou, China; ^3^ Clinic for Conservative and Preventive Dentistry, Center of Dental Medicine, University Zurich, Zurich, Switzerland; ^4^ Department of Applied Prosthodontics, Graduate School of Biomedical Sciences, Nagasaki University, Nagasaki, Japan

**Keywords:** abrasion, demineralized organic matrix, erosion, hollow mesoporous silica, quercetin, tubule occlusion

## Abstract

**Introduction:** Dental erosion and abrasion pose significant clinical challenges, often leading to exposed dentinal tubules and dentine demineralization. The aim of this study was to analyse the efficacy of quercetin-encapsulated hollow mesoporous silica nanocomposites (Q@HMSNs) on the prevention of dentine erosion and abrasion.

**Method:** Q@HMSNs were synthesized, characterized, and evaluated for their biocompatibility. A total of 130 dentine specimens (2 mm × 2 mm × 2 mm) were prepared and randomly distributed into 5 treatment groups (n = 26): DW (deionized water, negative control), NaF (12.3 mg/mL sodium fluoride, positive control), Q (300 μg/mL quercetin), HMSN (5.0 mg/mL HMSNs), and Q@HMSN (5.0 mg/mL Q@HMSNs). All groups were submitted to *in vitro* erosive (4 cycles/d) and abrasive (2 cycles/d) challenges for 7 days. The specimens in the DW, NaF, and Q groups were immersed in the respective solutions for 2 min, while treatment was performed for 30 s in the HMSN and Q@HMSN groups. Subsequently, the specimens were subjected to additional daily erosion/abrasion cycles for another 7 days. The effects of the materials on dentinal tubule occlusion and demineralized organic matrix (DOM) preservation were examined by scanning electron microscopy (SEM). The penetration depth of rhodamine B fluorescein into the etched dentine was assessed using confocal laser scanning microscopy (CLSM). The erosive dentine loss (EDL) and release of type I collagen telopeptide (ICTP) were measured. The data were analysed by one-way analysis of variance (ANOVA) with *post hoc* Tukey’s test (*α* = 0.05).

**Results:** Q@HMSNs were successfully synthesized and showed minimal toxicity to human dental pulp stem cells (HDPSCs) and gingival fibroblasts (HGFs). Q@HMSNs effectively occluded the dentinal tubules, resulting in a thicker DOM in the Q@HMSN group. The CLSM images showed more superficial penetration in the HMSN and Q@HMSN groups than in the quercetin, NaF, and DW groups. The Q@HMSN group exhibited a significantly lower EDL and reduced ICTP levels compared to the other groups (*p* < 0.05).

**Conclusion:** Q@HMSNs hold promise for inhibiting dentine erosion and abrasion by promoting tubule occlusion and DOM preservation.

## 1 Introduction

Oral diseases pose substantial public health challenges worldwide, with significant negative impacts on individuals and communities (Peres et al., 2019). Dental erosion, characterized by the chronic loss of dental hard tissues due to acid exposure and mechanical influences without bacterial involvement, has become prevalent, affecting a significant proportion of the population (Wiegand and Attin, 2007; Schlueter et al., 2011). Recent studies have indicated that dental erosion has affected 57.1% of European adults (Bartlett et al., 2013), with a global prevalence in permanent teeth of approximately 20% in children and 45% in adults (Schlueter and Luka, 2018), and this trend continues to grow (Vered et al., 2014). In the later stages of dental erosion, the opening of dentine tubules facilitates hypersensitivity and pulp infection (Aminoshariae and Kulid, 2021).

Dentine, which is primarily composed of collagen, plays a crucial role in dental structure, and preserving its demineralized organic matrix (DOM) is essential for preventing erosion (Mazzoni et al., 2015). However, the DOM is vulnerable to dissolution due to the presence of matrix metalloproteinases (MMPs) (Zarella et al., 2015). The application of MMP inhibitors has been demonstrated to effectively prevent and slow the disintegration of the DOM (Kato et al., 2010). Compounds such as quercetin, epigallocatechin gallate (EGCG), chlorhexidine (CHX), and proanthocyanidin (PA) have proven to be effective inhibitors of MMPs in dentine, protecting dental hard tissue against erosion (Balalaie et al., 2018). In particular, quercetin has demonstrated superior mechanical reinforcement after being crosslinked with collagen, emphasizing its potential clinical significance (Hong et al., 2022). However, the timing of inhibitor application was found to significantly affect the efficacy in terms of protecting against dentine erosion (Lapinska et al., 2018). In our previous study, the application of quercetin before acid attack resulted in a significantly thicker DOM compared to the application of well-known MMP inhibitors such as EGCG and CHX. However, when quercetin was applied after erosion, its effectiveness was notably reduced, and it could not protect the dentine tubule from exposure (Li et al., 2022). This reduced effectiveness is likely due to the surface targeting of collagen degradation and dissolution of the MMP inhibitors by the low pH environment following erosive attacks (Barbosa et al., 2019). Nevertheless, patients experiencing erosion often visit dental offices when the dentine has already been affected. Therapeutic interventions for treating dentine erosion frequently begin with treatment of the eroded tooth hard tissues, which usually involve exposed tubules. Consequently, appropriately managing quercetin on eroded tooth hard tissues may hold greater clinical significance than the pretreatment of intact dentine.

In recent decades, mesoporous silica nanoparticles (MSNs) have garnered attention for their well-structured framework, as they have a stable structure, chemical and thermal stability, a high pore volume, a large surface area, and excellent biocompatibility. MSNs have versatile applications as nanocarriers for drugs, proteins, genes, and enzymes (Izquierdo-Barba et al., 2009; Lee et al., 2016; Ni et al., 2018; Kankala et al., 2020; Zhang et al., 2021; Wang et al., 2022). In addition, the valuable role MSNs have in dental materials has been demonstrated by a growing body of literature, particularly in applications related to tubule occlusion and acid resistance (Chiang et al., 2014; Yu et al., 2016). Hollow MSNs (HMSNs) are of particular interest due to their mesoporous shells and hollow interiors (Li et al., 2017). The hollow cavities within HMSNs make them ideal carriers for drug delivery, particularly for compounds such as quercetin. In this context, using quercetin-encapsulated HMSNs (Q@HMSNs) presents a novel approach for addressing tooth erosion, showing the potential to block dentinal tubules after erosion and prevent dentine organic matrix breakdown. To our knowledge, no prior report is available on Q@HMSNs in the context of dental erosion, making this area an intriguing focus for research.

Therefore, the aim of the present study was to test the following null hypotheses: (1) Q@HMSNs have no effect on erosive dentine loss (EDL) after erosion and abrasion; (2) Q@HMSNs have no effect on dentine tubule occlusion after erosion and abrasion; and (3) Q@HMSNs have no effect on DOM preservation after erosion and abrasion.

## 2 Materials and methods

### 2.1 HMSNs synthesis

Silica nanoparticles (NPs) were synthesized using a modified Stöber method (Bai et al., 2019). Briefly, 6.28 mL of ammonium hydroxide solution (25%–28%; Aladdin, China), 42.8 mL of absolute ethanol (Xilong Scientific, China), and 20 mL of deionized water were mixed for 15 min under constant stirring, while 12 mL of tetraethyl orthosilicate (TEOS; Sigma, United States) was dissolved in 100 mL of ethanol. The latter solution was poured into the former solution under continuous stirring for 2 h (Mutlu et al., 2021). The SiO_2_ NPs were collected by centrifugation, deionized water was used to wash the sediment one time, and then the NPs were washed three times with ethanol. Subsequently, the SiO_2_ NPs was dried in an oven at 60°C overnight.

The SiO_2_ NPs were transformed into HMSNs using a self-templating strategy (Fang et al., 2013; Yu et al., 2021a). Five hundred milligrams of the SiO_2_ NPs were dispersed ultrasonically in 250 mL of deionized water. Then, 750 mg of cetyltrimethylammonium bromide (CTAB; Sigma, United States of America), 150 mL of ethanol, and 2.75 mL of ammonium hydroxide (25%–28%) were added, and the mixture was stirred continuously for 30 min at ambient temperature, after which TEOS (6 mL) was added and stirring continued for 6 h. After centrifugation, the sediment was redispersed in 100 mL of deionized water. Subsequently, 2.12 g of sodium bicarbonate (Na_2_CO_3_; Aladdin, China) was added, and the mixture was continuously stirred at 65°C for 24 h to, etch the hollow core. After centrifugation, the precipitate was washed twice with deionized water and ethanol, and the collected particles were calcined at 550°C for 6 h.

### 2.2 Q@HMSNs fabrication

One hundred milligrams of HMSNs was dispersed ultrasonically in 10 mL of ethanol. Then, 20 mg of quercetin (Sigma, United States) was added to the HMSNs solution with constant stirring at 400 rpm for 24 h. The mixture was then shaken at 150 rpm for an additional 24 h in the dark to achieve maximum quercetin loading. The suspension was washed with deionized water twice (Sapino et al., 2015; Saputra et al., 2022).

### 2.3 Q@HMSNs characterization

The morphologies of the HMSNs and Q@HMSNs were observed using transmission electron microscopy (TEM; JEM-F200, JEOL, Japan). Scanning TEM equipped with energy dispersive spectroscopy (EDS) was employed to examine the mapping patterns of elements. The crystalline phase was determined by applying X-ray diffraction (XRD; Smartlab 9KW, Rigaku, Japan). Small-angle and wide-angle XRD patterns were scanned from 0.5° to 10° and 10°–70°, respectively, and recorded in the 2θ range with a scanning speed of 1°/min. The functional groups in the sample were detected by Fourier transform infrared (FTIR) spectroscopy (iN10, Thermo Scientific, United States of America). The nitrogen adsorption–desorption isotherm was studied by an ASAP 2460M gas adsorption analyser (Micromeritics, United States of America) at 77 K. The pore size distribution, pore volume and surface area were calculated by the Brunauer–Emmett–Teller and Barrett–Joyner–Halenda (BET/BJH) methods. A Zetasizer Nano ZSP system (ZS90, Malvern Instruments, United Kingdom) was used to determine the size distribution. The effective amount of quercetin loaded into the HMSNs was determined using thermogravimetric analysis (TGA; TG 209 F3 Tarsus, NETZSCH, Germany) from 30°C to 1,000°C with a heating speed of 15°C/min.

### 2.4 Quercetin release from the Q@HMSNs

One hundred milligrams of Q@HMSNs was dispersed in 10 mL of phosphate-buffered saline (PBS; Xilong Scientific, China), and then the mixture was shaken at 120 rpm in the dark. The release of quercetin was measured at 0.5, 1, 2, 4, 8, 12, 24, 48, 72, 120 and 168 h. At each time point, the Q@HMSNs solution was centrifuged at 4,200 rpm for 4 min, and 1 mL of the liquid supernatant was collected for UV–Vis measurement (SpectraMax iD3, Molecular Devices, United States of America). The solution was then replenished with 1 mL of fresh PBS. The content of released quercetin was determined by measuring the absorbance at 377 nm (Yu et al., 2021b; Saputra et al., 2022).

### 2.5 Cytotoxicity evaluation

Human dental pulp stem cells (HDPSCs) and human gingival fibroblasts (HGFs) were taken from the pulp and peridental membranes of healthy adults who had given informed consent for third molar extraction, as approved by the Biomedical Research Ethics Committee of Local University (FMUSS 2018–20). α-Modified essential medium (α-MEM; HyClone, United States of America) with 1% penicillin/streptomycin (PS; Beyotime, China) and 10% foetal bovine serum (FBS; Gibco, Australia) were applied to culture the HDPSCs and HGFs in a humidified atmosphere of 5% CO_2_ at 37 °C. Subculturing was performed when the cells reached confluence. The HDPSCs and HGFs were collected at third passage and seeded in 96-well plates (2000 cells/well). After 24 h, the cells were cultured with complete medium containing Q@HMSNs extract solutions at concentrations of 1.0, 2.5, and 5.0 mg/mL. A control group without Q@HMSNs was included in the experiment. After an additional 1, 4, and 7 days of incubation, cell counting kit-8 (CCK-8; Dojindo Japan) (10 μL) was added to each well. Incubation was continued at 37°C for 2 h. Then, the absorbance at 450 nm was measured (SpectraMax iD3, Molecular Devices, United States). Each test was performed in triplicate, the relative viability rates of the HDPSCs and HGFs were calculated using the following equation (Xing et al., 2022).

$$\text{Relative cell viability }\left(\%\right)=\frac{{OD}_{Sample}-{OD}_{Medium}}{{OD}_{Control}-{OD}_{Medium}}\times 100\;\%$$

In addition, calcein/propidium iodide (PI) live/dead fluorescent dye (Beyotime, China) was utilized to stain the cells for 30 min on day 7. Images of cell morphology and proliferation were recorded by an inverted fluorescence microscope (Axio Observer A1, Carl Zeiss, Germany) (Huang et al., 2020).

### 2.6 Specimen preparation and experimental design

The study protocol was approved by the Institutional Review Board of the local university (2020QH2045), and all teeth were obtained after donors provided signed informed consent.

Seventy intact fresh human third molars were collected. All molars were immersed in a 0.5% thymol solution and stored at 4°C within 1 month of extraction prior to use. A low-speed saw (Buehler; Lake Bluff, IL, United States of America) was used to slice the molar to 145 dentine specimens (each 2 mm × 2 mm × 2 mm, the mid-crown portions beneath the enamel–dentinal junction) under water cooling. The dentine blocks were distributed for various analyses: 50 were taken for surface profile measurements (n = 10); 20 for surface morphology observations (n = 4); 25 for rhodamine B fluorescein evaluations (n = 5); and 50 for type I collagen telopeptide (ICTP) assessment (n = 10).

The dentine blocks were coupled to a customed silicone mould, embedded with acrylic resin (New century, China) and wet polished using silicon carbide sandpapers (#320, #600, #800, #1000, #1200, and #2000; Matador, Germany) for 60 s each. Subsequently, ultrasound was used to clean the surface of each specimen. The final dimensions of the specimens were as follows: 4 mm × 4 mm for the top surface, 5 mm × 5 mm for the bottom surface, and a thickness of 3 mm. Each specimen was covered on both sides with nail varnish (Top Speed; Revlon, United States), leaving a 1 mm × 1 mm area exposed as the erosion region (Jiang et al., 2020).

All samples were subjected to erosive and abrasive challenges (4 cycles/d) for 7 days to simulate an eroded tooth (Wiegand and Attin, 2011). In each erosion cycle, specimens were first immersed in artificial saliva (containing 0.795 g/L CaCl_2_·H_2_O, 0.4 g/L NaCl, 0.005 g/L Na_2_S·9H_2_O, 0.4 g/L KCl, 0.3 g/L KSCN, 1 g/L urea, and 0.69 g/L NaH_2_PO_4_·H_2_O, pH = 6.8) for 1 h (O Toole et al., 2015). Afterwards, the specimens were soaked in citric acid (pH = 2.45; Sigma, United States of America) for 5 min (Shellis et al., 2011), followed by a 10 s water rinse. Subsequently, the specimens were stored in renewed artificial saliva for 2 h before the next erosive challenge. Additionally, the specimens were brushed with a toothbrush for 2 min after the first and final daily erosive attacks (a total of 2 cycles per day) over a 7 days period, as previously described (Hong et al., 2020).

After the initial 7 days of erosive and abrasive challenges, the specimens were randomly divided into 5 groups (n = 26): DW (deionized water, negative control), NaF (12.3 mg/mL sodium fluoride, positive control), Q (300 μg/mL quercetin), HMSN (5.0 mg/mL HMSNs), and Q@HMSN (5.0 mg/mL Q@HMSNs).

**FIGURE 1:**
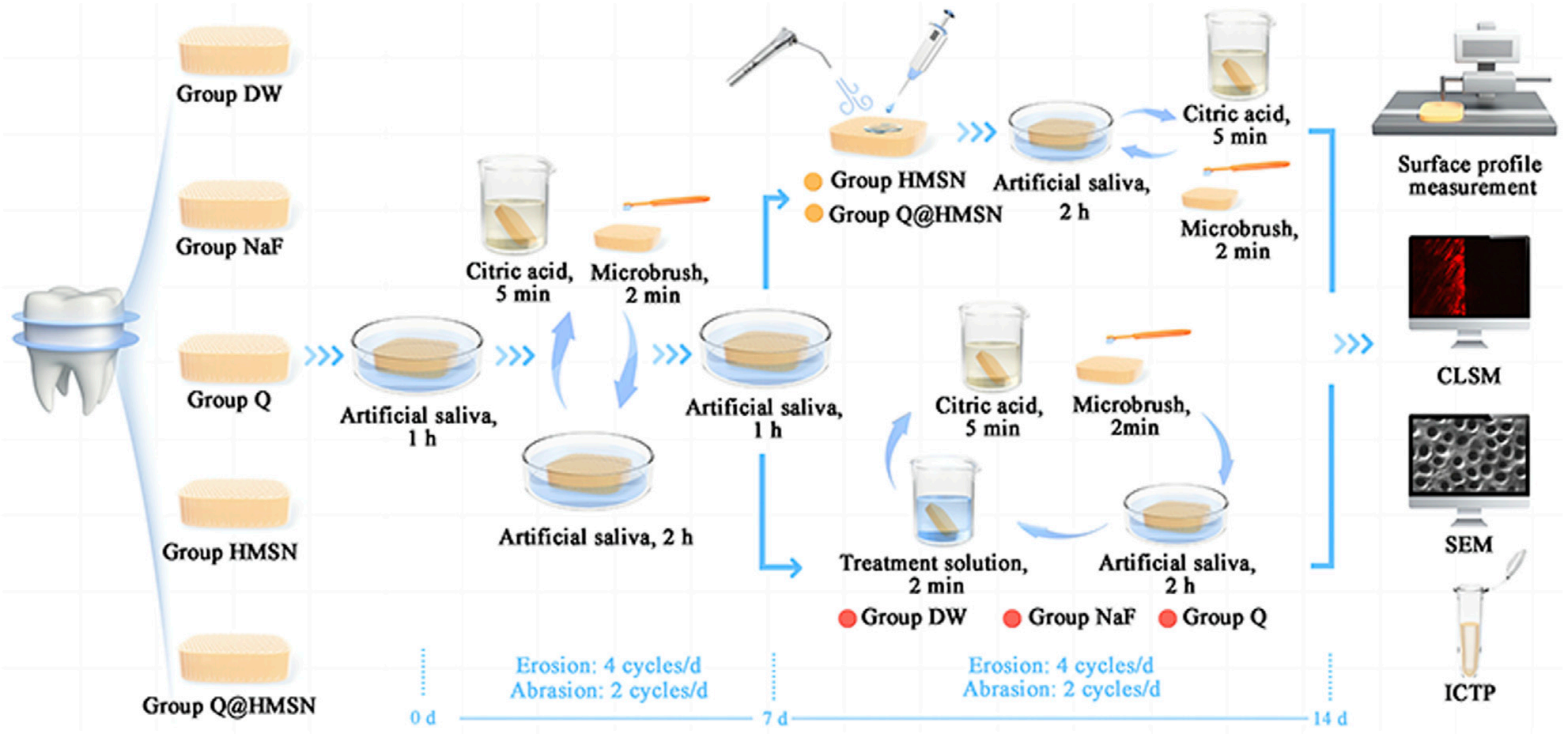
Flow chart of the study.

The samples in the DW, NaF, and Q groups were immersed in their respective solutions for 2 min each day, while the samples in the HMSN and Q@HMSN groups were treated as follows. First, 0.2 mL of the appropriate suspension was gently applied to the surface of the specimen, and then the surface was blown dry using a dental air‒water syringe for 30 s. Following these treatments, the specimens were placed in renewed artificial saliva for 2 h before undergoing daily cycles of erosion (4 cycles/d) and abrasion (2 cycles/d). The abrasion procedure, as described previously, was performed after the first and last erosive attacks over 7 days (2 cycles/d). However, unlike the DW, NaF and Q groups, the HMSN and Q@HMSN groups did not received additional treatment with HMSNs or Q@HMSNs during the subsequent 6 days of erosive and abrasive challenges (Figure 1).

### 2.7 Dentine morphology observations

Scanning electron microscopy (SEM; EM8000, KYKY, China) was employed to observe the morphology of the dentine surface after 7 and 14 days of erosive and abrasive challenges. Twenty specimens were placed in the freeze dryer for desiccation for 24 h and coated with gold via a sputter-coating process. Observations were made with both surface and cross-sectional views, with central site of each segment being examined at a ×5,000 magnification (Yu et al., 2017).

### 2.8 Surface profile measurements

The surface profiles of the specimens were measured after the 14 days experimental period using a contact profilometer (SEF680, Kosaka Laboratory, Japan) (Capalbo et al., 2022). Before evaluation, a scalpel blade was used to carefully remove the nail varnish. The EDL was calculated by determining the difference in height between the eroded surface and the reference surface. The surface of each sample was scanned with a stylus, and the mean value of 5 height difference measurements was recorded (in μm) as the EDL (Hannas et al., 2016).

### 2.9 Dentine permeability evaluation

Ten dentine specimens from each group (n = 2) were selected. Rhodamine B (0.1% (w/v)) was applied to the dentine surfaces of the specimens using a microbrush for 1 min. Afterwards, the specimens were washed with PBS three times. Subsequently, the specimens were longitudinally sectioned into slices, and confocal laser scanning microscopy (CLSM; FV3000, Olympus, Japan) was applied to observe the penetration depth of the specimens using laser excitation at 523 nm (Park et al., 2019).

### 2.10 Collagen degradation assessment

To evaluate the effect of the Q@HMSNs on the DOM, the release of ICTP was measured. The remaining 50 dentine specimens were demineralized with 10% phosphoric acid (pH = 1.0) (Aladdin, China) for 12 h at 25°C. Subsequently, the samples were rinsed in deionized water with continuous stirring at 4°C for 72 h, followed by drying in 60°C oven for 8 h. The specimens were then immersed in tubes contained 500 μL of artificial saliva at 37°C for 1 week. After centrifugation, the supernatants from each tube were collected, and an ELISA kit (MBbio, China) was used to analyse the release of ICTP (Osorio et al., 2011).

### 2.11 Statistical analysis

The data were analysed using the SPSS statistical software package (IBM, SPSS 19, United States of America). The equal variances and normality of the data were confirmed using the Levene test and the Kolmogorov–Smirnov test, respectively. For the CCK-8, EDL, and ICTP release assays, one-way analysis of variance (ANOVA) with *post hoc* Tukey’s test was used for data analysis. A value of *p* < 0.05 was considered to indicate statistical significance.

## 3 Results

### 3.1 Characterization of the Q@HMSNs

**FIGURE 2:**
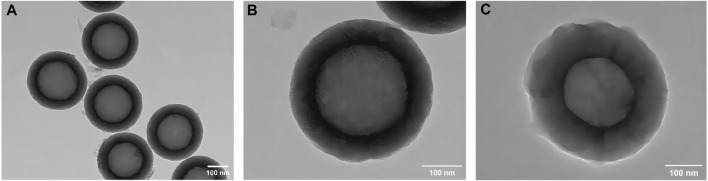
TEM images of **(A,B)** HMSNs and **(C)** quercetin-encapsulated HMSNs. HMSNs, hollow mesoporous silica nanocomposites.

The TEM image of the HMSNs in Figures 2A, B revealed spherical NPs with an approximate diameter of 400 nm and a well-ordered channel framework shell structure with a cavity. After loading quercetin, the mesoporous structure remained intact, albeit slightly obscured, indicating that quercetin had been successfully incorporated into the HMSNs with maintenance of the overall morphology (Figure 2C). The mapping pattern of scanning TEM/EDS was used to identify the element distribution. The presence of Si and O signals was demonstrated in both the HMSNs and Q@HMSNs, with the intensity of C in Q@HMSNs being further highlighted (Supplementary Figure S1).

**FIGURE 3:**
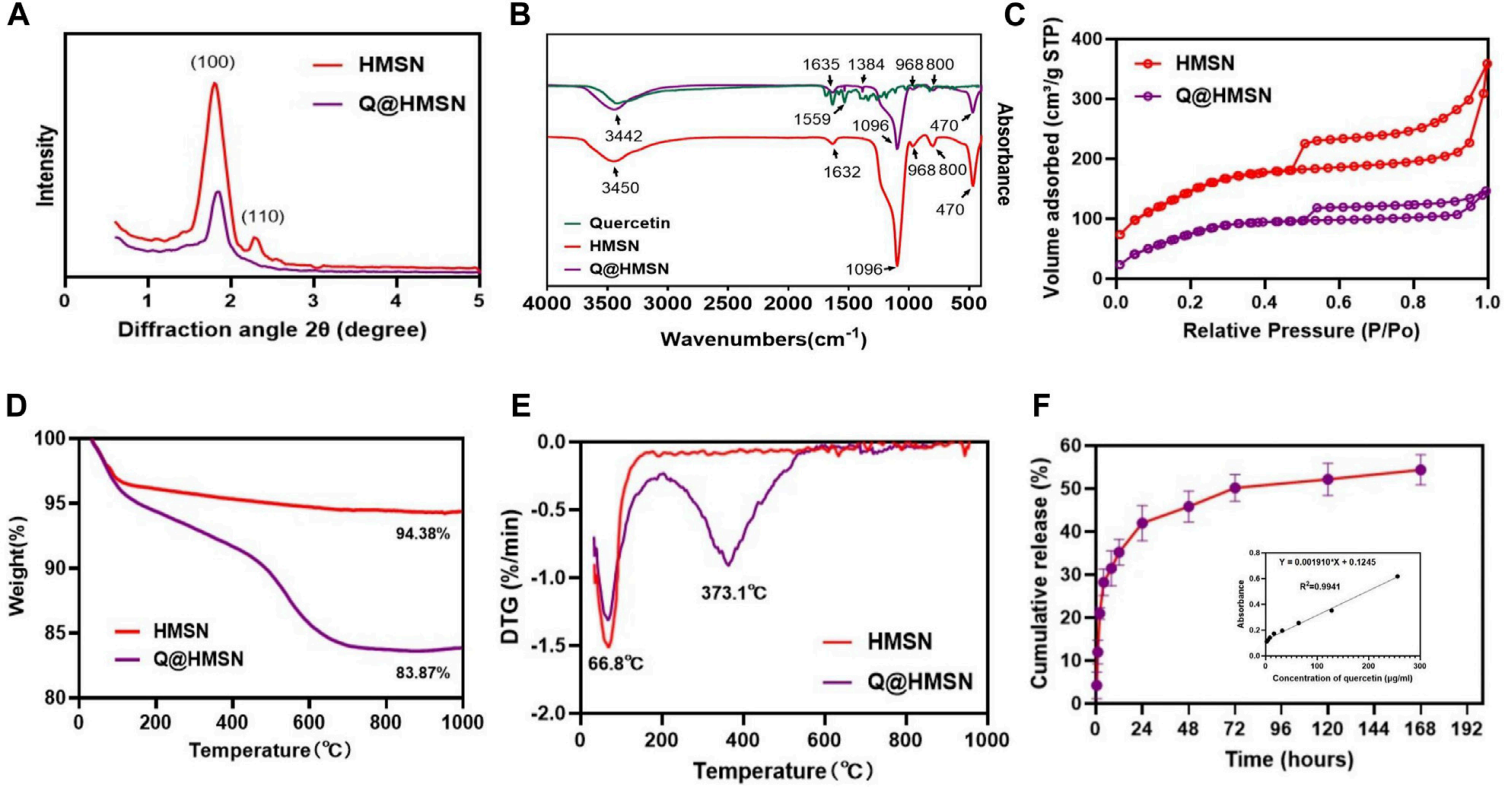
Characterization of the HMSNs and Q@HMSNs. **(A)** Small-angle XRD, **(B)** FTIR spectra, **(C)** nitrogen adsorption–desorption isotherms, **(D)** thermogravimetric analysis, **(E)** derivative thermogravimetric analysis, and **(F)** release profile of quercetin from the Q@HMSNs. HMSNs, hollow mesoporous silica nanocomposites; Q@HMSNs, quercetin-encapsulated hollow mesoporous silica nanocomposites.

The mesoporous crystalline phase and structure were analysed using XRD. For the small-angle pattern (Figure 3A), the XRD analysis of the HMSNs displayed a characteristic diffraction peak at 2θ = 1.9° (100), consistent with a mesoporous structure. Notably, the intensity of this diffraction peak decreased after quercetin loading. In the wide-angle pattern (Supplementary Figure S2), both the HMSNs and Q@HMSNs presented a broad band of non-crystalline scattering around 2θ = 22°. The Q@HMSNs did not exhibit any crystalline peaks in the observed 2-theta range. This could be explained by the quercetin changing into a non-crystalline state (Yu et al., 2023).

Additionally, in the FTIR spectrum of the HMSNs, distinctive peaks at 968 cm^−1^ (Si–OH bending vibration), 1,096 cm^−1^ (Si–O–Si vibration), 800 cm^−1^, 470 cm^−1^ (Si–O stretching and bending vibration), and 1,632 cm^−1^ (H–O–H bending vibration) were observed. When compared to the HMSNs spectrum, the spectrum of the Q@HMSNs exhibited changes due to quercetin loading at 1,635 cm^−1^ (C=O stretching vibration), 1,559 cm^−1^, 1,384 cm^−1^ (C=C stretching vibrations) and 3,442 cm^−1^ (OH stretching) (Elmowafy et al., 2022). The presence of these functional groups confirmed the successful loading of quercetin (Figure 3B).

Nitrogen absorption-desorption isotherms were used to characterize the mesoporous characteristics of both the undoped HMSNs and Q@HMSNs. Both the HMSNs and Q@HMSNs presented type IV isotherms, and their mesoporous structure curves were in accordance with the TEM images and XRD analysis. The specific surface area (SBET), pore volume (V_P_), and pore diameter (D_P_) of the HMSNs, calculated using the BET/BJH method, were found to be 597.5 m^2^/g, 0.567 cm^3^/g, and 3.93 nm, respectively. After quercetin loading, the SBET and V_P_ decreased to 391.8.9 m^2^/g and 0.242 cm^3^/g, respectively. This reduction indicated the successful loading of quercetin into the mesopores of the silica NPs (Figure 3C; Supplementary Figures S3, S4; Supplementary Table S1).

TGA and derivative thermogravimetric analysis (DTG) were employed to assess the quantity and thermal stability of the quercetin loaded into the HMSNs. TGA revealed a weight loss of 5.62 wt% for the HMSNs and 16.13 wt% for the Q@HMSNs (Figure 3D). The prominent downwards peak at 66.8°C in the DTG curve corresponds to the evaporation of physically adsorbed water, while another significant downwards peak at 373.1°C indicates the decomposition of the organic substance quercetin. These results confirmed the encapsulation of quercetin into the HMSNs, with a calculated loading amount of 10.51 wt% (Figure 3E; Supplementary Figure S5).

Figure 3F; Supplementary Figure S6 illustrates the drug release profile from the Q@HMSNs, which indicated an initial burst release during the first 8 h, followed by a gradual slowing of the release rate, finally resulting in sustained release over 7 days.

### 3.2 Biocompatibility evaluation

**FIGURE 4:**
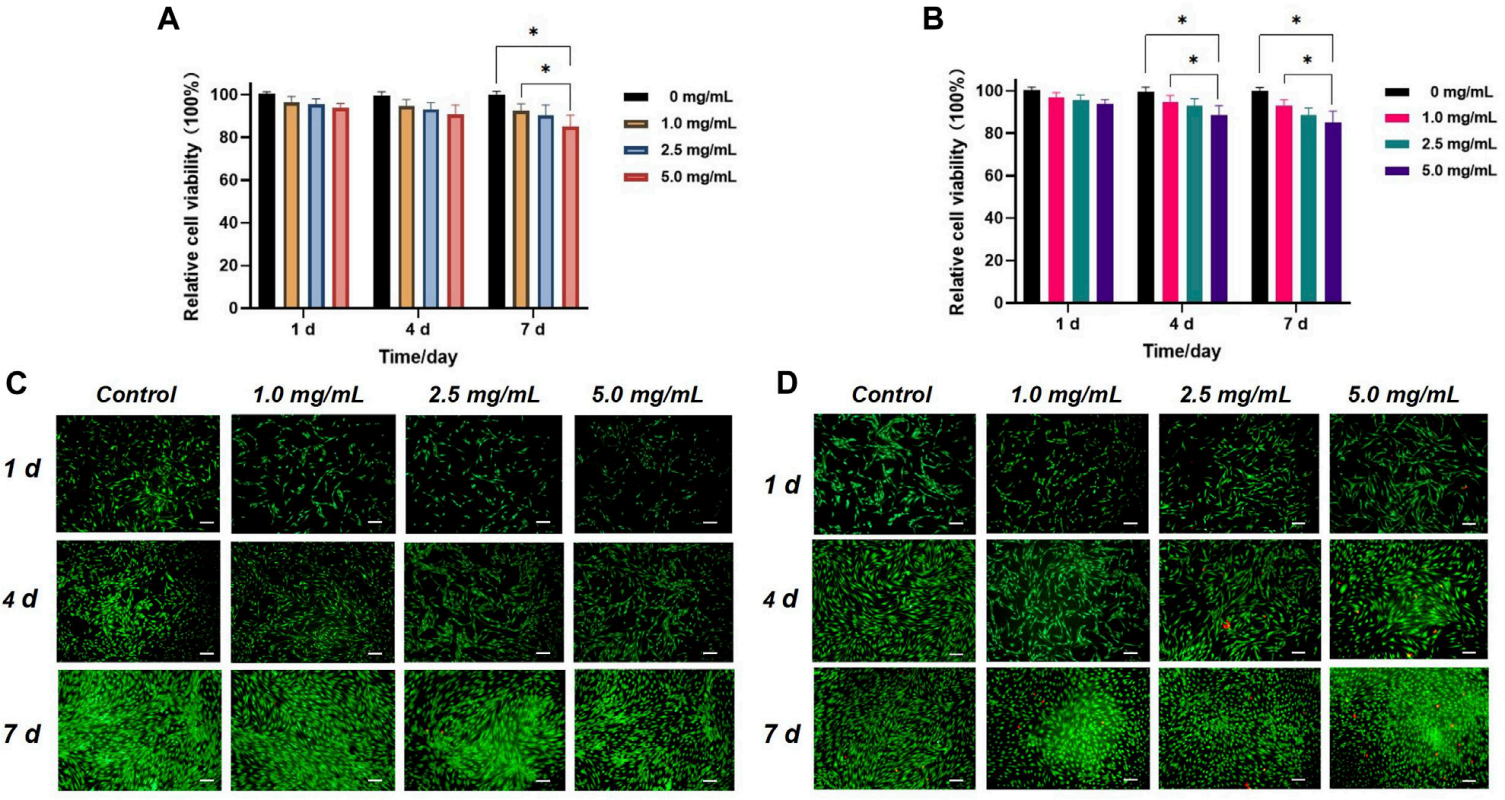
*In vitro* biocompatibility evaluation of the Q@HMSNs. **(A)** Results of the CCK-8 assay with human gingival fibroblasts at 1, 4, and 7 d *p < 0.05. **(B)** Results of the CCK-8 assay with human dental pulp stem cells at 1, 4, and 7 d *p < 0.05. **(C)** Calcein/PI live/dead fluorescence staining images of human gingival fibroblasts incubated with different concentrations of Q@HMSNs. **(D)** Calcein/PI live/dead fluorescence staining images of human dental pulp stem cells incubated with different concentrations of Q@HMSNs. The data are presented as means and standard deviations. Scale bar: 20 µm. HMSNs, hollow mesoporous silica nanocomposites; Q@HMSNs, quercetin-encapsulated hollow mesoporous silica nanocomposites.

Figures 4A, B depict the relative viabilities of HGFs and HDPSCs after exposure to different concentrations of Q@HMSNs (1.0, 2.5, and 5.0 mg/mL) for 1, 4, and 7 days. These figures illustrate that even at the highest concentration of 5.0 mg/mL, the viabilities of HDPSCs and HGFs exceeded 80% after 7 days of incubation. The live/dead fluorescence staining results of HGFs and HDPSCs in Figures 4C, D further illustrate the substantial cell proliferation at each concentration, with minimal induction of cell death by Q@HMSNs compared to the other groups. These results provide strong evidence that Q@HMSNs induced relatively negligible toxicity to HGFs and HDPSCs.

### 3.3 Tubule occlusion and DOM preservation

**FIGURE 5:**
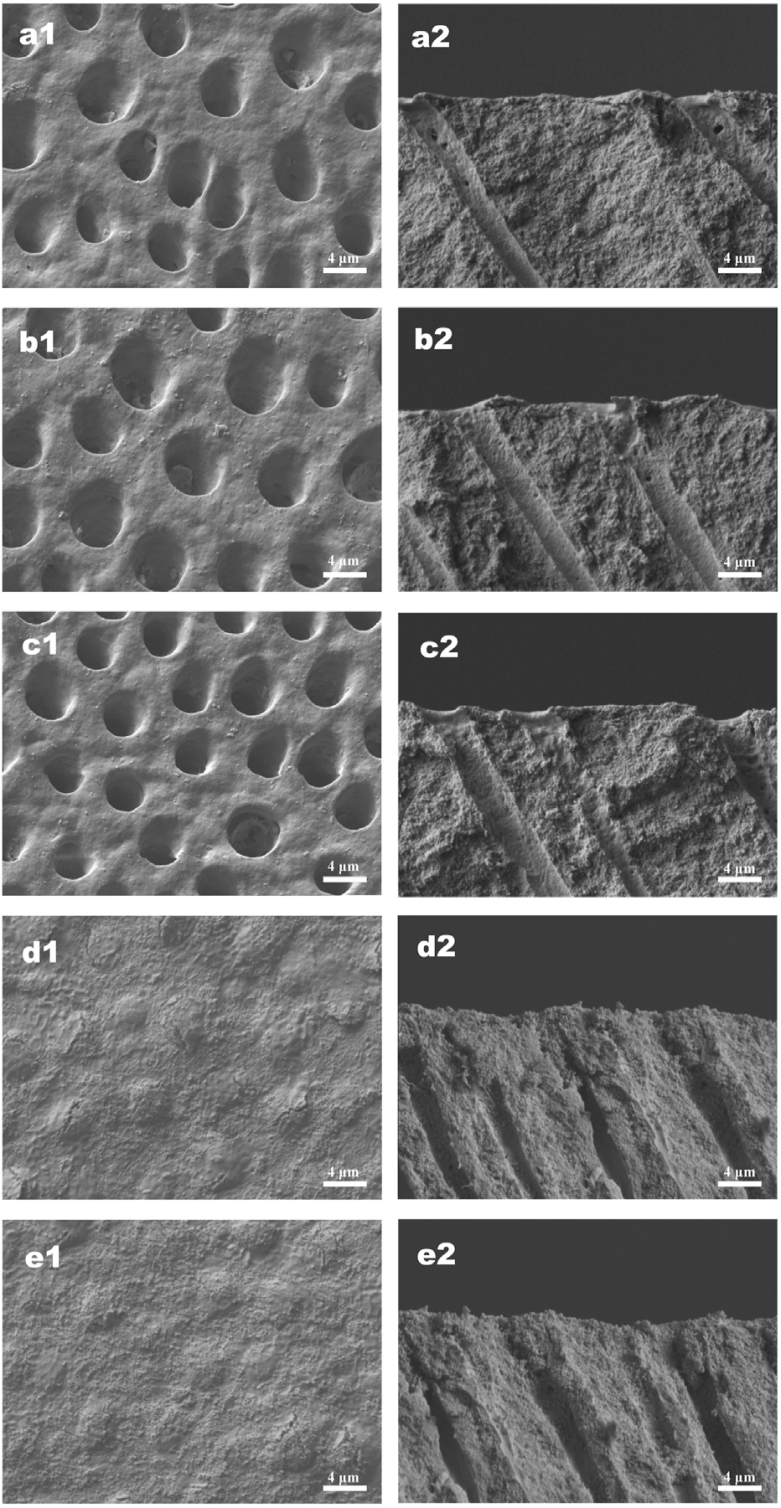
Representative SEM images (×5,000 magnification) of specimens treated with the respective solutions immediately after 7 days of erosion and abrasion. **(a1−e1)** Corresponding surface-section SEM images of specimens. **(a2−e2)** Corresponding cross-section SEM images of specimens. **(a1,a2)** Specimens treated with deionized water. **(b1,b2)** Specimens treated with NaF. **(c1,c2)** Specimens treated with quercetin. **(d1,d2)** Specimens treated with HMSNs. **(e1,e2)** Specimens treated with Q@HMSNs. The images in d1, d2, e1, and e2 show that the dentine tubules were occluded by HMSNs and Q@HMSNs. HMSNs, hollow mesoporous silica nanocomposites; Q@HMSNs, quercetin-encapsulated hollow mesoporous silica nanocomposites.

**FIGURE 6:**
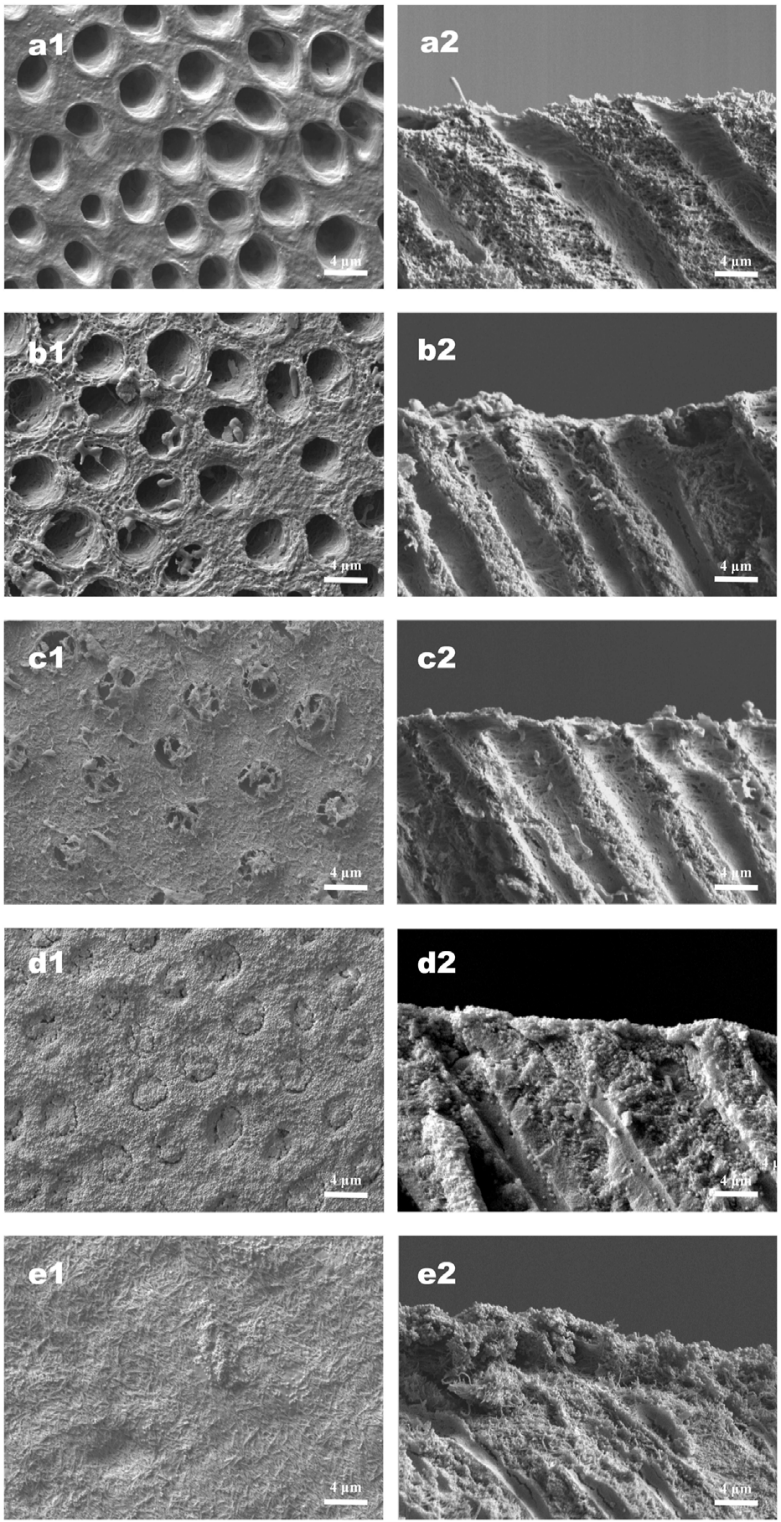
Representative SEM images (×5,000 magnification) of specimens under continuous erosive and abrasive challenge for 14 d **(a1−e1)** Corresponding surface-section SEM images of specimens. **(a2−e2)** Corresponding cross-section SEM images of specimens. **(a1,a2)** Specimens treated with deionized water. **(b1,b2)** Specimens treated with NaF. **(c1,c2)** Specimens treated with quercetin. **(d1,d2)** Specimens treated with HMSNs once. **(e1,e2)** Specimens treated with Q@HMSNs once. The images in d1, d2, e1, and e2 show clear dentine tubule occlusion, and the images in e1 and e2 show a notably distinct and thick DOM surface. HMSNs, hollow mesoporous silica nanocomposites; Q@HMSNs, quercetin-encapsulated hollow mesoporous silica nanocomposites.

The effects of different treatments on tubule occlusion and DOM preservation are presented in Figures 5, 6. After the application of HMSNs and Q@HMSNs, the dentinal tubules in the HMSN and Q@HMSN groups were completely obstructed. The cross-sectional images confirmed that these tubes were effectively sealed by the NPs. In contrast, the tubules in the DW, NaF, and Q groups were exposed due to acid attack (Figure 5). After 14 days of erosion and abrasion, the DW and NaF groups showed mostly exposed tubules. Moreover, narrower tubules were observed in group Q, while the tubules were blocked in the HMSN group. In contrast, the specimens treated with Q@HMSNs demonstrated well-defined dentine tubule occlusion with integration of the DOM and microparticles. Moreover, the cross-sectional view shows a notably distinct and thick DOM in the Q@HMSN group compared with the other groups (Figure 6).

### 3.4 Dentine tubule permeability

**FIGURE 7:**
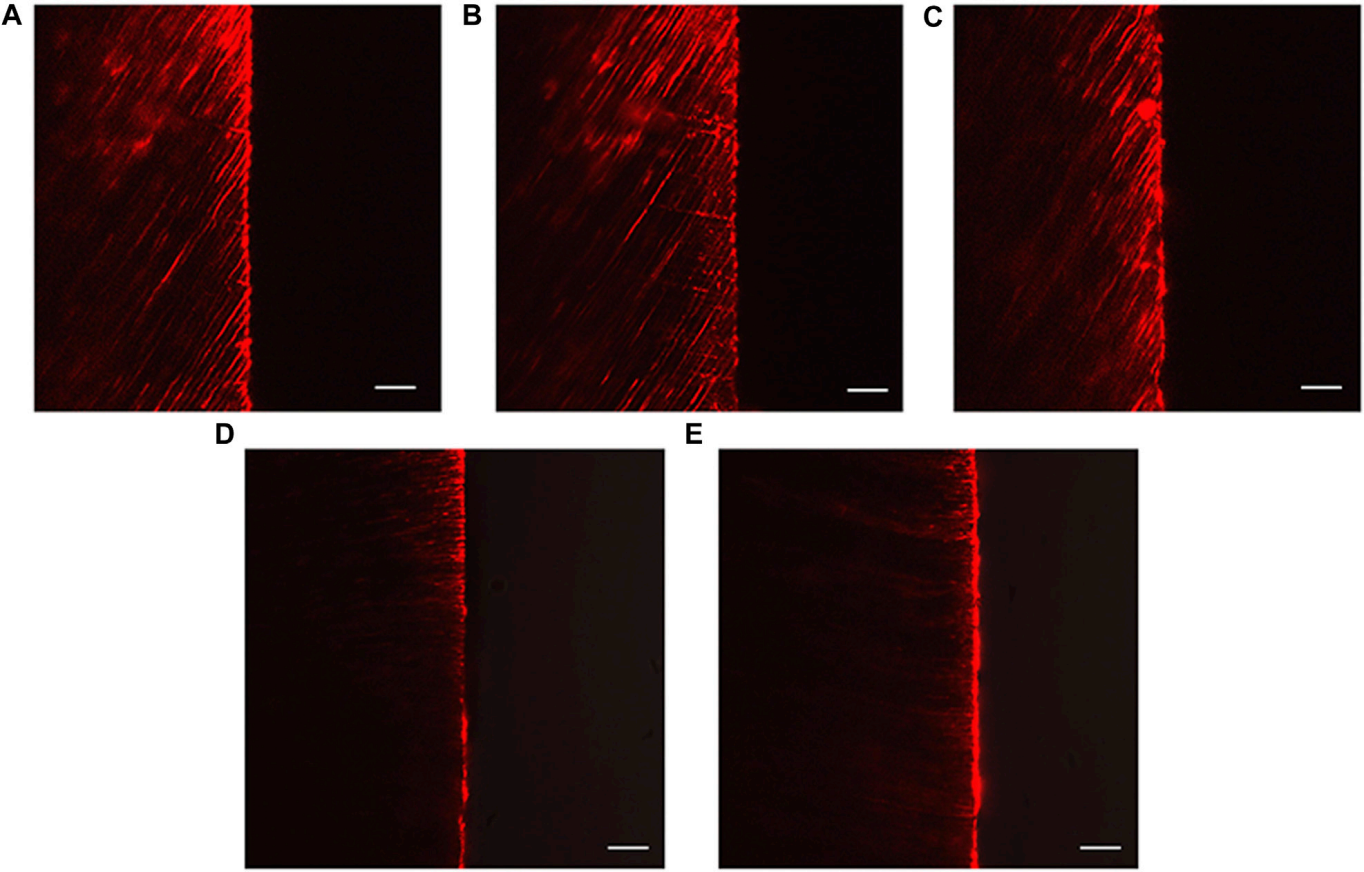
Confocal images of vertically sectioned specimens that were treated with the respective solutions after 14 days of erosion and abrasion. **(A)** Specimens treated with deionized water. **(B)** Specimens treated with NaF. **(C)** Specimens treated with quercetin. **(D)** Specimens treated with HMSNs. **(E)** Specimens treated with Q@HMSNs (magnification, ×40, scale bar: 20 µm). HMSNs, hollow mesoporous silica nanocomposites; Q@HMSNs, quercetin-encapsulated hollow mesoporous silica nanocomposites.

The CLSM images revealed the penetration depth of rhodamine B in each group. The DW, NaF, and Q groups showed extensive rhodamine B penetration into the dentinal tubules (Figures 7A–C). In contrast, superficial fluorescent bands were observed in the HMSN and Q@HMSN groups (Figures 7D, E).

### 3.5 Effect of Q@HMSNs on EDL

**FIGURE 8:**
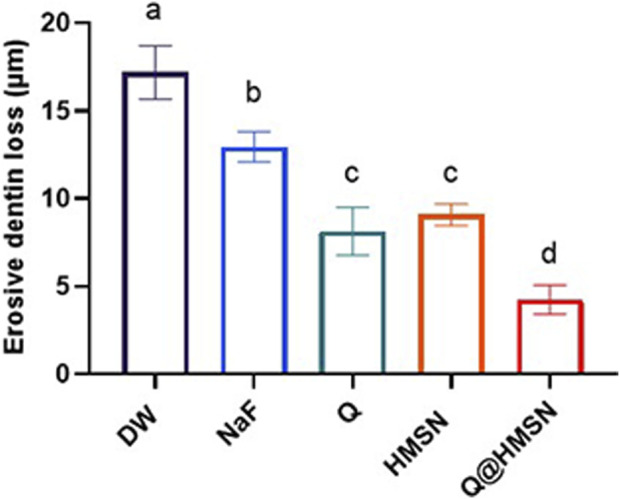
Means and standard deviations of the erosive dentine loss values in each group. Values marked with the same superscript letter were not significantly different (*p* > 0.05).

Figure 8 shows the EDL of the specimens treated with different solutions. Significantly less EDL was observed in the NaF, Q, HMSN and Q@HMSN groups than in the DW group (*p* < 0.05). The Q@HMSN group had the lowest EDL among the tested groups (*p < 0.05*).

### 3.6 Effect of Q@MSNs on collagen release

The results from ICTP analysis revealed that 44.34 ± 3.37, 40.13 ± 4.38, and 43.35 ± 3.43 g/L ICTP were released in the DW, NaF, and HMSN groups, respectively, with no significant differences among these values (*p >* 0.05). In contrast, ICTP release was significantly reduced in the Q and Q@HMSN groups (19.37 ± 4.38 and 12.36 ± 2.49 g/L, respectively) (*p < 0.05*).

## 4 Discussion

In this study, the potential of using Q@HMSNs as dentinal tubule sealing agents and their efficacy to inhibit dentine erosion and abrasion were investigated. The findings supported effective dentinal tubule sealing, DOM preservation, and reduced EDL following acid erosion and abrasion and treatment with Q@HMSNs, leading to the rejection of all three null hypotheses. The erosion challenge model was utilized to simulate the impact of dietary soft drinks, with 4 erosion cycles/d and 2 abrasion cycles/d (Shellis et al., 2011). A concentration of 12.3 mg/mL sodium fluoride (NaF), a widely studied treatment for tooth erosion, was chosen as the positive control (Magalhaes et al., 2010). Moreover, 300 μg/mL quercetin was chosen based on established protocols (Jiang et al., 2020). Based on previous studies (Hong et al., 2022), the effects of quercetin on dentin erosion may be related to the inhibition of dentin-derived MMPs and the enhancement in the mechanical properties of dentin collagen fibre. In the present study, collagen degradation was performed using ICTP ELISA kits. The present results showed that Q@HMSNs significantly affected the activation of dentin-derived MMPs. The primary inhibitory mechanism of Q@HMSNs on MMPs can be attributed to the inefficiency of MMPs due to the quercetin blocking their bonding with Zn^2+^/Ca^2+^ (Saragusti et al., 2010). Additionally, the phenolic hydroxyl groups in quercetin can form stable hydrogen bonds with hydroxyl group in collagen (Zhai et al., 2010).

Historically, various approaches, such as calcium-containing pastes (Pei et al., 2013), sodium fluoride (Anderson et al., 2020), and laser treatment (Meng et al., 2023), have been used for tubule obstruction, but limitations persist, including shallow infiltration and the susceptibility to re-exposure during acid attack (Wang et al., 2010). Beltrame et al. (2018) reported a 28% reduction in EDL with phosphorylated chitosan after 5 days of acid erosion. Li et al. (2022) reported that the EDL decreased by 45% after quercetin was applied after acid attacks. Schlueter et al. (2009) reported that the EDL was reduced approximately 29% after the application of NaF and 7 days of citric acid demineralization compared to the placebo. In the present study, the effects of HMSNs and Q@HMSNs surpassed these results, with HMSNs providing a 47% reduction in EDL and Q@HMSNs delivering a 75% reduction.

Considering its unique tubular structure, mesoporous silica has been employed as an effective biomimetic vehicle for dentine (Yu et al., 2021a). TEM images of the Q@HMSNs confirmed their stable framework and the successful incorporation of quercetin into the MSNs. Nitrogen adsorption–desorption analysis demonstrated a type IV isotherm for the HMSNs and Q@HMSNs, indicating a mesoporous structure. The *in vitro* release profiles demonstrated that the HMSNs served as a reservoir and carrier for sustained quercetin delivery. The CCK-8 assay results revealed the low cytotoxicity of the Q@HMSNs, ensuring their suitability for gingival fibroblast and dental pulp cell proliferation. The dentine permeability assessments and SEM observations indicated enhanced erosion and abrasion resistance 14 days after treatment with the HMSNs and Q@HMSNs. Q@HMSNs showed potential as a durable system against acid attacks, with hollow mesoporous silica exhibiting a superior loading capacity compared to traditional mesoporous silica (Elmowafy et al., 2022). Additionally, the quercetin loaded into the HMSNs may be more resistant to degradation inside the tubules than on the dentine surface. Even after a single application to the dentine specimens, the Q@HMSNs demonstrated significant tubule occlusion and DOM preservation capabilities.

Due to the advantageous properties of NPs, such as MSNs and bioactive glass NPs (BGNs), these materials have been widely employed to efficiently occlude dentinal tubules and maintain stability against everyday acid erosion (Li et al., 2018; Zhang et al., 2018; Jung et al., 2019a; Jung et al., 2019b). However, few studies have explored the combined effect of MSNs with MMP inhibitors on the DOM. In this study, Q@HMSNs demonstrated dual functionality by occluding tubules and protecting the DOM from acid erosion. The EDL results indicated that using Q@HMSNs was more effective than applying HMSNs separately. In a study by Vertuan et al. (2021), preservation of the DOM reduced the EDL by 28% compared to conditions without the DOM. Viana et al. (2020) used β-tricalcium phosphate (β-TCP) NPs to combat dental erosion, resulting in an approximately 50% reduction in the EDL compared to the control group. While the HMSN group in our study showed a 47% reduction in EDL, the Q@HMSN group demonstrated a higher reduction (75%). This suggests that the presence of Q@MSNs may lead to favourable tubule occlusion, providing enhanced protection for quercetin against acid attack. With the dissolution of dentine mineralization and release of quercetin, the remaining dentine organic matrix combines with Q@HMSNs to form a membrane-like layer (Figures 6e1, e2). Based on the present findings, we assume that the main mechanism of action for inhibiting dentine erosion and abrasion is that Q@MSNs could efficiently occlude the dentinal tubules and protect DOM. Theoretically, the HMSNs, as antiacid inorganic contents on the surface dentin, will prevent the further exposure of dentine tubules and demineralization of dentine. Beyond that, quercetin holds inhibitory activity against MMPs, strengthens the mechanical properties of dentin collagen, and maintains the organic matrix on the erosion surface. Moreover, the Q@HMSNs demonstrated high biocompatibility and the potential for use in *in vivo* applications. The continuous quercetin release over 7 days and sustained anti-erosion ability highlight the clinical potential of Q@HMSNs.

This study has a few limitations. Firstly, the efficacy of the Q@HMSNs was observed *in vitro*, and further studies are needed to explore its *in situ*/*in vivo* applications. Additionally, this study did not include CHX, EGCG, or other MMP inhibitors. Future studies should assess and compare the effectiveness of other MMP inhibitors encapsulated in HMSNs.

## 5 Conclusion

Despite these limitations, the results here suggest that Q@HMSNs could effectively inhibit dentine erosion and abrasion through tubule occlusion and DOM preservation. Q@HMSNs hold promise as an innovative measure for treating dentine erosion and abrasion in future *in vivo* applications.

## Data Availability

The raw data supporting the conclusion of this article will be made available by the authors, without undue reservation.
