# Supplementary material for: Enhancing the inhibition of dental erosion and abrasion with quercetin-encapsulated hollow mesoporous silica nanocomposites
Source: Front Bioeng Biotechnol. 2024 Feb 8;12:1343329. doi: 10.3389/fbioe.2024.1343329 (PMC10885352; doi:10.3389/fbioe.2024.1343329)
Supplement: Supplementary file 1 [file DataSheet1.docx]

Supporting Information

Enhancing the inhibition of dental erosion and abrasion with quercetin-encapsulated hollow mesoporous silica nanocomposites

**Jia-min Chen^1,†^****, Yi-ling Cheng^1,†^, Meng-hui Yang^1^, Chen Su^1^, Hao Yu^1,2,3,4,*^**

^1^Fujian Key Laboratory of Oral Diseases & Fujian Provincial Engineering Research Center of Oral Biomaterial & Stomatological Key Laboratory of Fujian College and University, School and Hospital of Stomatology, Fujian Medical University, Fuzhou, China

^2^Department of Prosthodontics, School and Hospital of Stomatology, Fujian Medical University, Fuzhou, China

^3^Clinic for Conservative and Preventive Dentistry, Center of Dental Medicine, University Zurich, Switzerland

^4^Department of Applied Prosthodontics, Graduate School of Biomedical Sciences, Nagasaki University, Nagasaki, Japan

*** Correspondence:**

Hao Yu

[haoyu-cn@hotmail.com](mailto:haoyu-cn@hotmail.com)

**Keywords: abrasion, demineralized organic matrix, erosion, hollow mesoporous silica, quercetin, tubule occlusion**

Jia-min Chen and Yi-ling Cheng contributed equally to this work.

**Figures S1 to S6**

**Tables S1**


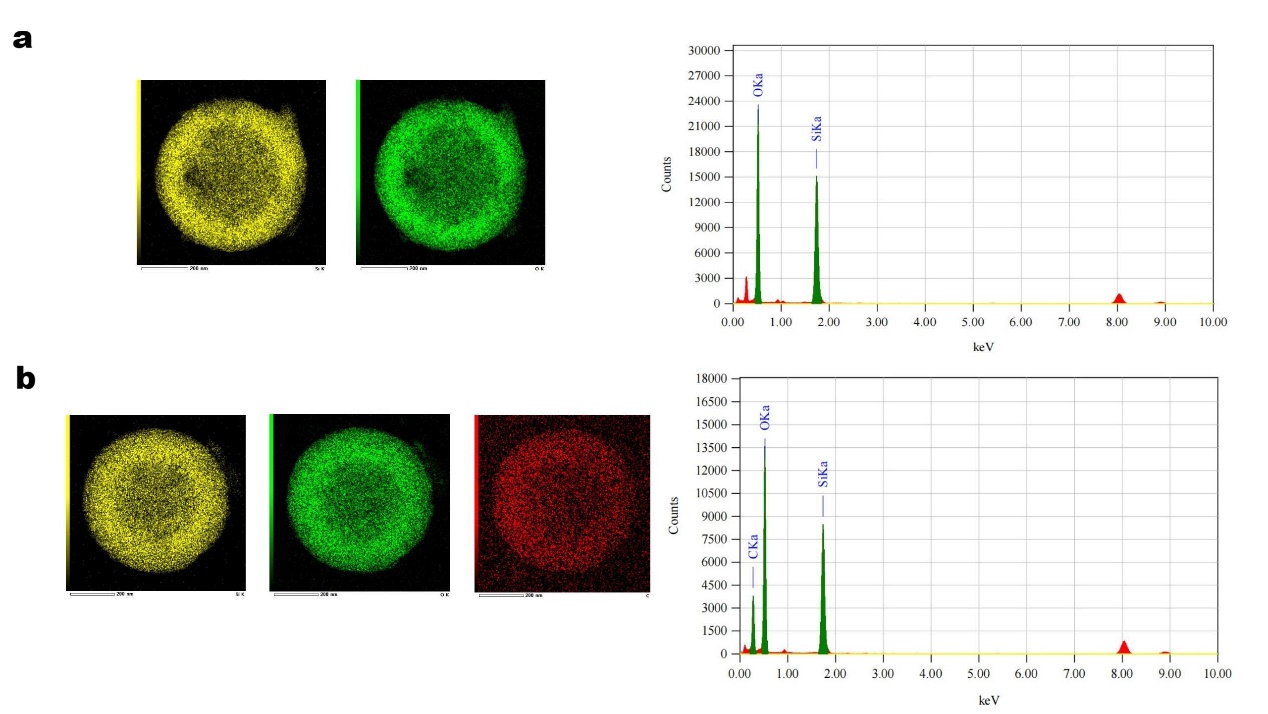


**Figure S1.** Scanning TEM-EDS elemental mapping patterns of (a) HMSN and (b) Q@HMSN, HMSN showing distributions of Si (yellow) and O (green) signals, Q@HMSN showing distributions of Si (yellow), O (green) signals and C (red) signals.


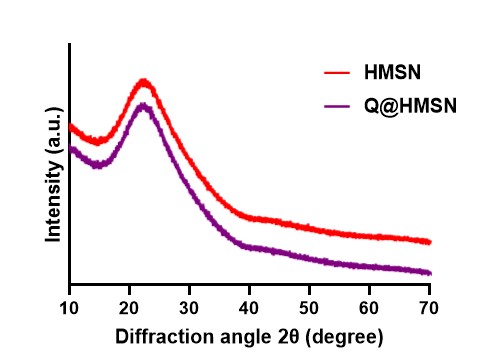


**Figure S2.** Wide-angle XRD spectra of HMSN and Q@HMSN.


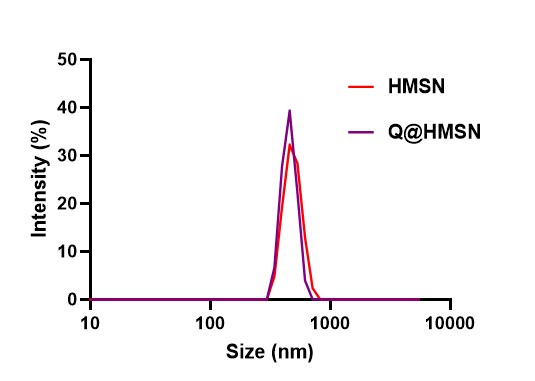


**Figure S3.** Size distributions of HMSN and Q@HMSN.


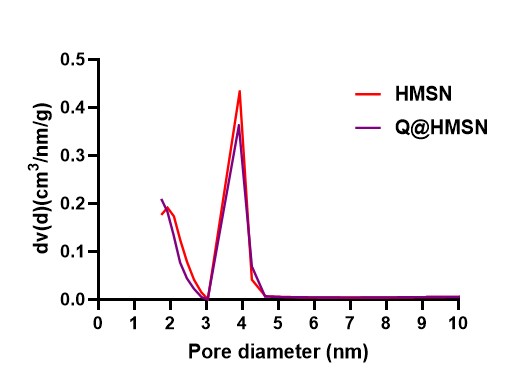


**Figure S4.** Pore size distribution of HMSN and Q@HMSN.


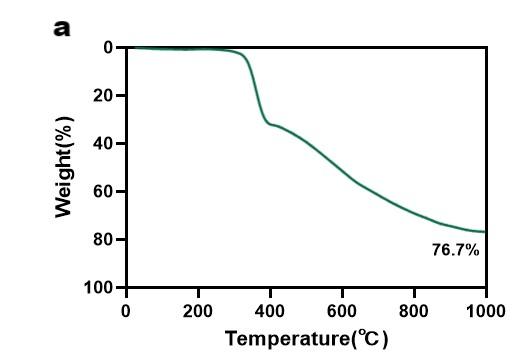

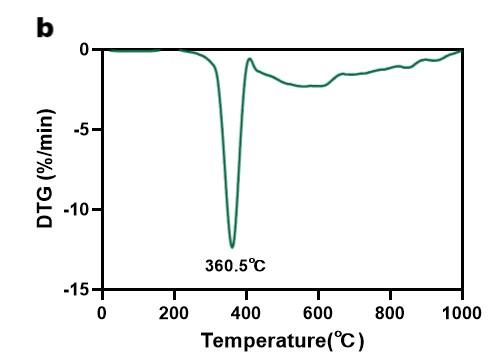


**Figure S5.** thermogravimetric analysis (a) and derivative thermogravimetric analysis (b) of quercetin.


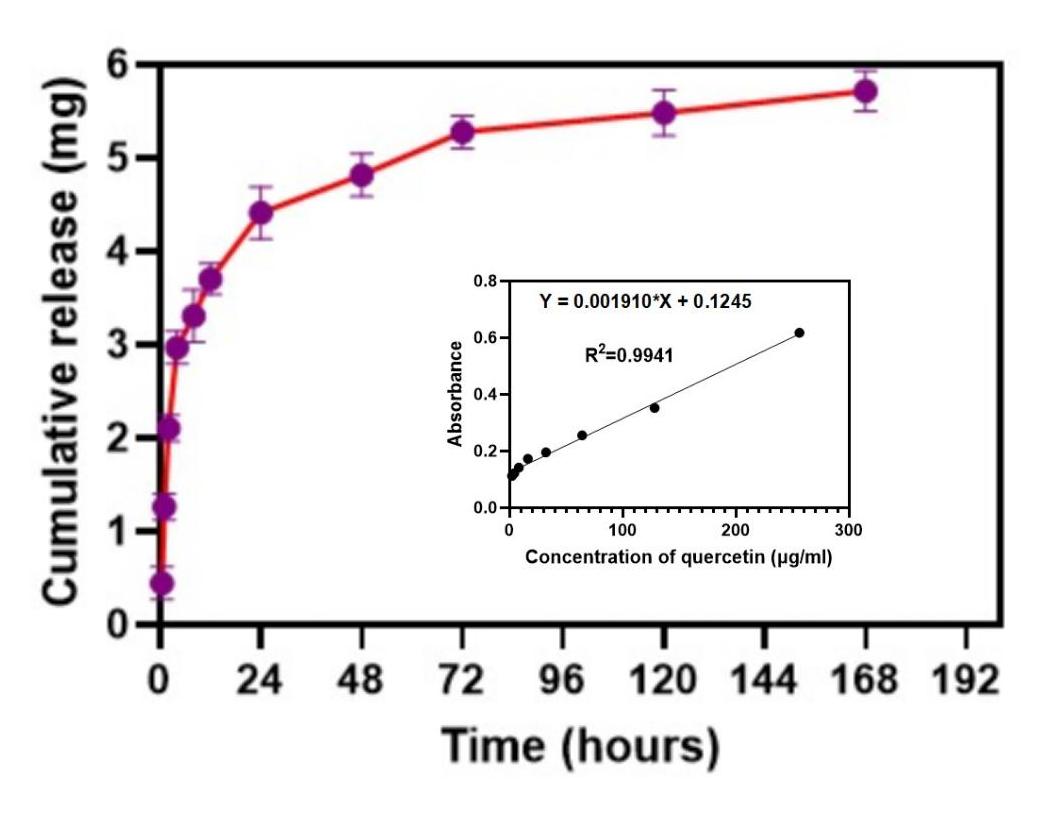


**Figure S6.** The release profile of quercetin from the Q@HMSNs.

**Table S1.** Summary of physico-chemical characteristics of HMSN and Q@HMSN.
